# Evaluation of a Novel Missense Mutation in *ABCB4* Gene Causing Progressive Familial Intrahepatic Cholestasis Type 3

**DOI:** 10.1155/2020/6292818

**Published:** 2020-06-15

**Authors:** Komal Saleem, Qingbo Cui, Tahir Zaib, Siqi Zhu, Qian Qin, Yusi Wang, Jinxi Dam, Wei Ji, Peng Liu, Xueyuan Jia, Jie Wu, Jing Bai, Songbin Fu, Wenjing Sun

**Affiliations:** ^1^Laboratory of Medical Genetics, Harbin Medical University, Harbin 150081, China; ^2^Key Laboratory of Preservation of Human Genetics Resources and Disease Control in China (Harbin Medical University), Ministry of Education, China; ^3^Pediatric Surgery, The Second Affiliated Hospital of Harbin Medical University, China; ^4^Michigan State University, USA

## Abstract

Progressive familial intrahepatic cholestasis type 3 (PFIC3) is a hepatic disorder occurring predominantly in childhood and is difficult to diagnose. PFIC3, being a rare autosomal recessive disease, is caused by genetic mutations in both alleles of *ABCB4*, resulting in the disruption of the bile secretory pathway. The identification of pathogenic effects resulting from different mutations in ABCB4 is the key to revealing the internal cause of disease. These mutations cause truncation, instability, misfolding, and impaired trafficking of the MDR3 protein. Here, we reported a girl, with a history of intrahepatic cholestasis and progressive liver cirrhosis, with an elevated gamma-glutamyltransferase level. Genetic screening *via* whole exome sequencing found a novel homozygous missense mutation *ABCB4*:c.1195G>C:p.V399L, and the patient was diagnosed with PFIC3. Various computational tools predicted the variant to be deleterious and evolutionary conserved. For functional characterization studies, plasmids, encoding *ABCB4* wild-type and selected established mutant constructs, were expressed in human embryonic kidney (HEK-293T) and hepatocellular carcinoma (HepG2) cells. *In vitro* expression analysis observed a reduced expression of mutant protein compared to wild-type protein. We found that *ABCB4* wild type was localized at the apical canalicular membrane, while mutant p.V399L showed intracellular retention. Intracellular mistrafficking proteins usually undergo proteasomal or lysosomal degradation. We found that after treatment with proteasomal inhibitor MG132 and lysosomal inhibitor bafilomycin A1, MDR3 expression of V399L was significantly increased. A decrease in MDR3 expression of mutant V399L protein may be a result of proteasomal or lysosomal degradation. Pharmacological modulator cyclosporin A and intracellular low temperature (30°C) treatment significantly rescued both the folding defect and the active maturation of the mutant protein. Our study identified a novel pathogenic mutation which expanded the mutational spectrum of the *ABCB4* gene and may contribute to understanding the molecular basis of PFIC3. Therefore, genetic screening plays a conclusive role in the diagnosis of rare heterogenic disorders like PFIC3.

## 1. Introduction

Progressive familial intrahepatic cholestasis type 3 (PFIC3) is a subclass of heterogenic PFIC, a rare autosomal recessive liver disorder. It typically occurs in infancy and childhood, beginning with persistent cholestasis that progresses to cirrhosis and liver failure before late childhood [1, 2]. Pathology of disease is characterized by ductular proliferation in the liver and progressive intrahepatic cholestasis with elevated gamma-glutamyltranspeptidase (GGT) activity. The basic genetic defect of PFIC3 is characterized by reduced secretion of phosphatidylcholine (PC) into bile, which in turn impaired the bile secretory transport system [3, 4]. Reduced PC secretion causes toxicity in the liver and results in the destruction of hepatocytes that further progresses to intrahepatic liver cirrhosis.

PFIC3 patients are generally homozygous, heterozygous, or compound heterozygous for *ABCB4* mutations. Biallelic mutation of human *ABCB4*, located on chromosome 7q21.1 encoding lipid floppase MDR3 protein, is involved in causing PFIC3 [5–7]. MDR3 is primarily expressed at the canalicular membrane of the liver and acts as a phospholipid translocator, i.e., phosphatidylcholine (PC). It protects the hepatocyte membrane from detergent activity of bile salts [8]. MDR3 is involved in the floppase activity of phospholipids, transferring PC molecules from inner to outer canalicular membrane of hepatocytes [9–11]. *In vitro* studies demonstrated that the absence of PC floppase activity leads to impaired transport to the canalicular membrane, stops its binding with bile salts, and destabilizes mixed micelles. It can cause solubilization of the apical membrane and the hepatobiliary epithelium by detergent action of free bile salts, which induces inflammation and cell death of liver cells [12, 13].

Previous studies support the evidence that clinical signs and pathological findings of PFIC3 are nonspecific which makes diagnosis difficult in one-third of children with PFIC3 [14]. It is difficult to diagnose rare diseases without the use of genetic screening and analysis [15]. Diagnosis is based on liver histology with ductular proliferation, high level of liver enzymes, GGT, and bile acid concentrations. PFIC3 patients can be treated with ursodeoxycholic acid (UDCA), a hydrophilic bile acid that only recovers the symptoms in nearly 30% of cases. In severe cases, the ultimate alternative is liver transplantation [5]. Previously, it has been reported that ivacaftor (VX-770) could also be used for the treatment of such patients with the *ABCB4* defective mutation [16]. Evidence from animal models also demonstrated that disruption of MDR3 function results in progressive liver cirrhosis [9, 17, 18]. Recently, it has been found that hydrophilic tetrahydroxylated bile acids (THBA) have hepatoprotective functions in mice and could stop the progressive liver pathology associated with the Mdr2^−/−^ mutation [17].

In the present study, we reported a 13-year-old girl with a history of cholestasis, progressive liver cirrhosis, and an abnormal liver function of unknown etiology. Through clinical and genetic screening, the patient was later diagnosed with PFIC3. We aimed to identify the pathogenic variant and determine the pathogenicity of the variant by using *in vitro* molecular assays.

## 2. Materials and Methods

### 2.1. Clinical Description

**Figure 1:**
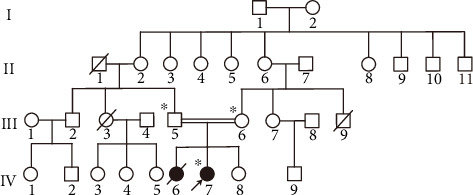
Family tree of affected family. Pedigree of a Chinese family with progressive familial intrahepatic cholestasis type 3.

In this study, a four-generation family was recruited (Figure 1) (Harbin, China). The pedigree shows consanguinity between the proband's parents. The proband had two sisters. The elder one was diagnosed with liver cirrhosis and hepatosplenomegaly at the age of 7 and died at 10 years old. The younger sister is normal and is 9 years old. The proband is a 13-year-old girl who has been diagnosed with cholestatic liver and related severe symptoms. Clinical information and peripheral blood were obtained from IV-7 (proband), III-5 (father), and III-6 (mother). The study protocol was approved by the Institutional Research Board of Harbin Medical University and conducted in accordance with the Declaration of Helsinki. All the participants provided signed informed consent.

### 2.2. Pathogenic Gene Detection

Peripheral blood was collected into a qualified anticoagulant EDTA tube. Genomic DNA extraction was performed using the DNeasy Blood & Tissue Kit (Qiagen, #69506, Germany) according to the manufacturer's protocol. In order to find all the possible pathogenic mutations, whole exome sequencing (WES) of the proband (IV-7) was conducted by Novogene Co., Ltd. (Beijing, China).

### 2.3. Variant Analysis

A novel mutation, *ABCB4*:NM_000443.3:exon11:c.G1195C:p.V399L, was found in the next-generation sequencing data. Based on targeted exome-based next-generation sequencing, primer sequences used for PCR and DNA sequencing of the candidate gene *ABCB4* (exon 11) were designed (supplementary table 1). PCR was performed with a final volume of 50 *μ*l. Under cycling condition, the first step (denaturation) was performed at 95°C for 10 mins. The sample was then subjected to 30 cycles of 30-second denaturation (at 95°C), 30 seconds of annealing at 55°C, and 30 seconds of extension at 72°C. The reaction was terminated and was followed by a final extension for 5 minutes at 72°C. Mutation analysis was confirmed based on the Sanger sequencing results and the NCBI (http://www.ncbi.nlm.nih.gov/genbank/) transcript sequence *ABCB4*:NM_000443.3, used as a reference to describe the nucleotide substitution.

### 2.4. Mutagenesis, Cell, and Transfection

The plasmid pReceiver-M02 (GeneCopoeia, Maryland, USA) containing a full open reading frame of *ABCB4:*NM_000443.3 was used as a template for introducing V399L and other established missense mutations (S346I, T424A, I541F, and R652G) *via* site-directed fast mutagenesis system (Trans BioNovo, Beijing, China). Primers were designed according to kit protocol to generate site-specific mutations (supplementary table 2). All *ABCB4* mutant constructs were verified by Sanger sequencing (supplementary Fig 1).

Human embryonic kidney HEK-293T cells and hepatocellular carcinoma HepG2 cells (from ATCC, Manassas, USA) were seeded onto poly-L-lysine-coated six-well plates at a density of 3.5 × 10^5^ cells/well 24 hrs before transfection. Plasmid vectors encoding *ABCB4* wild type and mutants (S346I, V399L, T424A, I541F, and R652G) were transiently transfected into HEK-293T and HepG2 cells, respectively, using JetPRIME reagent (Polyplus-transfection, Illkirch, France) following the manufacturer's protocol. To examine whether mutant protein underwent proteasomal or lysosomal degradation as described previously [19], cells were treated with 10 *μ*M MG132 (proteasomal inhibitor) or 0.1 mg/ml bafilomycin A_1_ (lysosomal inhibitor) after 24 hrs of transfection. Cells were treated with 10 *μ*M cyclosporin A after 6 hrs of transfection to check the effect of cyclosporin A on intracellular retained mutant. To check if lowering temperature can rescue mutant protein translocation, cells were incubated at 30°C after transfection. Cyclosporin A, bafilomycin A_1_, and MG132 were purchased from Sigma-Aldrich (Darmstadt, Germany).

### 2.5. Immunoanalysis

For immunofluorescence, HEK-293T and HepG2 cells were seeded on glass coverslips in six-well plates for 24 hrs before transfection. After 48 hrs of transfection, cells were fixed and permeabilized with 4% paraformaldehyde for 20 min at room temperature, followed by PBS-T washing for 10 min at 4°C and PBS-B (1% BSA) blocking for 30 min. Cells were incubated overnight with anti-MDR3 P3II-26 antibody (1 : 100) (CAT#: AM32042SU-N, OriGene Technologies, Rockville, USA) at 4°C, followed by 1 hr incubation with anti-mouse AlexaFluor594-conjugated antibody (Molecular Probes, Eugene, USA) at room temperature. The nucleic acid was stained using DAPI. Images were developed *via* a Leica DM5000B confocal laser scanning microscope (Leica Microsystems, Solms, Germany).

For immunoblotting, cells were lysed in ice cold lysis buffer after 48 hrs of transfection. Protein concentration was checked *via* BCA assay (Applygen Technologies, Beijing, China). Lysates were separated by 7.5% (*w*/*v*) SDS–PAGE and transferred to polyvinylidene difluoride membrane, followed by incubation of blots with anti-MDR3 P3II-26 antibody (1 : 1,000) and anti-mouse antibody (1 : 10,000) (Rockland Immunochemicals, Gilbertsville, PA). Immunoblotting of *β*-actin with a monoclonal antibody (Invitrogen, UK) was also performed as a reference control. The signal was obtained *via* the Odyssey CLx-imaging system (Li-COR, Lincoln, USA). The intensity of bands was measured using ImageJ software (National Institutes of Health, Bethesda, USA).

### 2.6. Quantitative Reverse Transcription-Polymerase Chain Reaction (qRT-PCR)

Total RNA was extracted *via* TRIzol reagent (Invitrogen, UK) according to the manufacturer's protocol. The first strands of complementary DNAs (cDNA) were synthesized by reverse transcription of 2 *μ*g of total RNA using the Transcriptor First Strand cDNA Synthesis Kit (Roche Applied Science, Alameda, USA). Transcribed cDNA was used as a template for qRT-PCR. Using ACTB as a reference gene, qRT-PCR was performed in triplicate by the LightCycler FastStart DNA Master SYBR Green and LightCycler detection system (Roche Applied Science). The primers used for qRT-PCR were human ABCB4: 5′-GAGAGGACACAAACCAGACAGCA-3′ (forward); 5′-GTCTGCCCACTCTGCACCTT-3′ (reverse); human ACTB: 5′-CAGAAGGATTCCTATGTGG-3′ (forward); 5′-CATGATCTGGGTCATCTTC-3′ (reverse). The relative quantitative expression was calculated *via* the 2^−*ΔΔ*Ct^ method.

### 2.7. Statistical Analysis

Statistical significance was determined using one-way analysis of variance (*ANOVA*) and Tukey's Multiple Comparison Test. *P* < 0.05 was considered as statistically significant.

### 2.8. In Silico Analysis

The frequency of variant was assessed in the 1000 Genome Project (TGP) (http://www.1000genomes.org) and the gnomAD (https://gnomad.broadinstitute.org/). Based on the American College of Medical Genetics and Genomics (ACMG) guidelines, pathogenicity of the variant was interpreted. The potential significance of the mutant variant was predicted using Mutation Taster (http://www.mutationtaster.org/), PolyPhen-2 (http://genetics.bwh.harvard.edu/pph2/), SIFT (http://sift.bii.a-star.edu.sg), PROVEAN (http://provean.jcvi.org/seq_submit.php), and MutPred2 (http://mutpred.mutdb.org/). Evolutionary conservation was checked by Aminode (http://www.aminode.org/) and UCSC (http://genome.ucsc.edu/), and the multiple sequence alignment was edited by Jalview software. In addition, NetPhos3.1 server was used for the prediction of any change in the alteration of phosphorylation profile (http://www.cbs.dtu.dk/services/NetPhos/). A display score of 0.5 was selected as a threshold. For each residue (i.e., threonine, serine, and tyrosine), only the best predictions with a display score ≥ 0.5 were considered phosphorylated.

## 3. Results

### 3.1. Clinical Description of Patient Diagnosed with PFIC3

The proband, the second child of the consanguineous couple, was born with normal delivery. She was presented with a history of cholestasis and severe liver malfunction at the age of 7. Further investigation for disease occurrence showed no family history of liver disease. Parents and the younger sister (9 years old) were normal without any evidence of cholestatic liver disease while one older sister died of the same disease at 10 years old. Comprehensive physical examination of the proband revealed mild jaundice, while ultrasound reports revealed cholecystitis, gallstone, liver cirrhosis, ascites, and hepatosplenomegaly. Overall, the pedigree of this family presented an autosomal recessive pattern of inheritance (Figure 1).

Laboratory test results at the time of admission revealed that total bilirubin, direct bilirubin, reticulocytes, gamma-glutamyltransferase (GGT), alanine aminotransferase (ALT), alkaline phosphatase (ALP), aspartate aminotransferase (AST), and bile acids in the plasma were elevated (Supplementary table 3). Complete blood count (CBC) revealed anemia with low hemoglobin level and reticulocytes. Based on clinical data and other laboratory details related to elevated serum GGT, the patient was later diagnosed with PFIC3. Immunohistochemical staining for the patient was not done because of the unavailability of liver tissue.

### 3.2. Variant Analysis of ABCB4:c.1195G>C Missense Mutation Found in Whole Exome Sequencing

**Figure 2:**
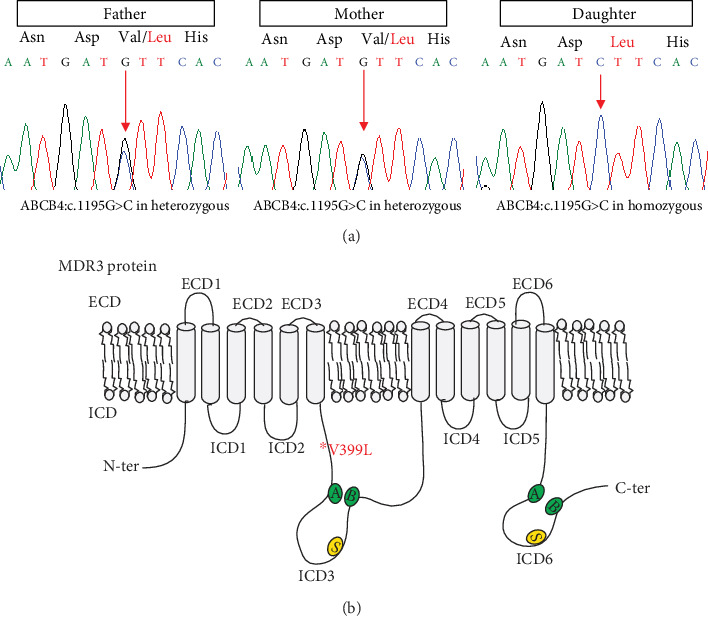
Structural and genetic analysis of *ABCB4*. (a) Sanger sequencing results showed a homozygous nucleotide change in the patient at nucleotide position 1195 from guanine to cytosine (c.1195G>C), leading to an amino acid change from valine to leucine at position 399 of MDR3 (p.V399L). Both parents were heterozygous for this mutation. (b) Structural domains of MDR3 depicting p.V399L mutation near first walker A motif of intracellular domain 3 (ICD3).

To identify the underlying causative gene, we performed whole exome sequencing of the proband. Genetic screening identified a novel homozygous missense mutation c.1195G>C in exon 11 of *ABCB4* at chr7: 87073014 (GRCH37/hg19), causing substitution of valine (GTT) to leucine (CTT) at amino acid position 399. To ascertain the mutation in the proband, targeted DNA fragments from the proband, her mother, and father were analyzed by bidirectional Sanger sequencing. Sanger sequencing results indicated that the proband was homozygous for the missense variant (*ABCB4*:c.1195G>C). Homozygosity pattern was also confirmed by the identification of the same heterozygous mutation in both parents (Figure 2(a)). The missense variant identified in WES data was defined as *ABCB4*:c.G1195C:p.V399L (NM_000443.3) and located in ICD3 domain near the first walker A motif of MDR3 protein (Figure 2(b)).

### 3.3. Missense Variant ABCB4:c. 1195G>C Was Predicted to Be Deleterious by In Silico Analysis

Various *in silico* analysis tools were utilized, and a novel mutation *ABCB4*:c.G1195C:p.V399L was predicted as “pathogenic.” PolyPhen-2 with a score of 0.886 predicted the variant to be possibly damaging. The SIFT result showed that novel mutation p.V399L was predicted to affect protein function with a score of 0.003. Mutation taster also predicted the mutant variant as disease causing with a probability value of 0.99997. MutPred2 interpreted the predictive score of 0.543 (54%) for V399L (higher score reflects higher probability of pathogenicity). PROVEAN predicted the mutation to be neutral. Furthermore, multiple sequence alignment of sequences, edited with the help of Jalview [20], showed that novel mutation *ABCB4*:c.1195G<C:p.V399L was physicochemically and evolutionarily conserved in most mammals (Supplementary Fig. 2). In addition, netphos3.1 server predicted that this mutation did not disturb the transport activity of protein by phosphorylation change at the posttranslational level. The missense variant, *ABCB4*:c.1195G<C:p.V399L, was not found in human variation databases (1000 genomes Project, dbSNP, EVS, gnome AD). It also was not registered in the Human Gene Mutation Database (HGMD) (http://www.hgmd.cf.ac.uk/ac/index.php) or ClinVar (http://www.ncbi.nlm.nih.gov/clinvar). Up to our knowledge, *ABCB4*:c.1195G<C:p.V399L was therefore considered as a novel mutation.

### 3.4. Subcellular Localization of MDR3 Protein Showed Intracellular Retention of ABCB4:c.1195G>C:p.V399L Mutant

**Figure 3:**
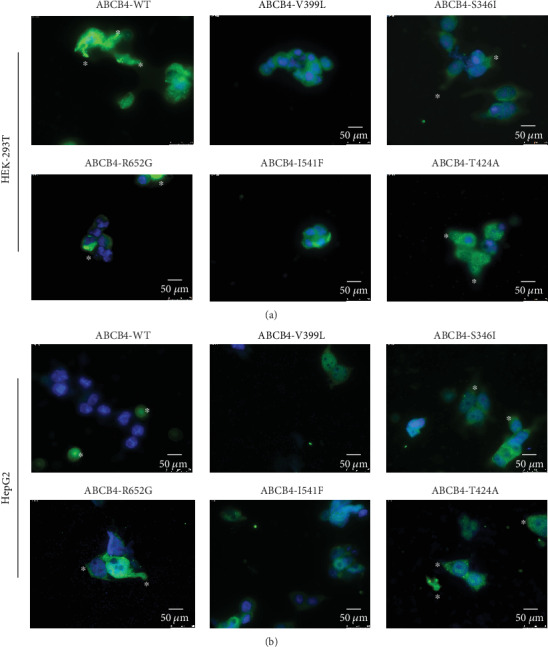
Immunofluorescence expression of *ABCB4* wild type and mutants in HEK293T cells. (a) HEK-293T cells and (b) HepG2 cells expressing *ABCB4* wild type and mutants were fixed with 4% paraformaldehyde and processed for immunofluorescence using anti-MDR3 antibody and anti-mouse AlexaFluor594-conjugated secondary antibody. DAPI was used for nuclei staining. Asterisk signs indicate bile canaliculi displaying apical localization. Bars indicate 50 *μ*m.

To examine the mechanism through which *ABCB4* mutations affect translocation, we checked MDR3 expression levels and differences between subcellular localization of wild-type and mutant proteins on the plasma membrane using immunofluorescence. We assumed that because of V399L mutation, protein may have altered intracellular retention, which would prevent MDR3 from properly functioning. Forty-eight hours after transfection, immunofluorescence in both HEK-293T and HepG2 cells showed that MDR3 wild type was completely expressed on the apical plasma membrane. MDR3 expression of three comparative mutants S346I, T424A, and R652G was also found to be localized and expressed on the plasma membrane-like MDR3 wild type. However, we observed that MDR3 expression of V399L, just like I541F [21], was absent on the plasma membrane and showed intracellular retention (Figures 3(a) and 3(b)).

### 3.5. The Expression of ABCB4:c.1195G>C:p.V399L Was Reduced

**Figure 4:**
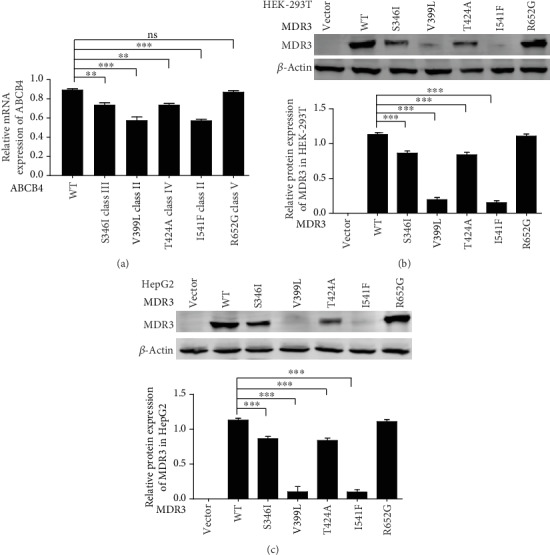
Expression analysis of ABCB4 wild type and mutants. (a) qRT-PCR expression analysis. (b) Western blot analysis in HEK-293T cell and (c) HepG2 cells. Three independent Western blot analyses effectively showed the same results. ns: nonsignificant, ^∗∗^*P* < 0.01, ^∗∗∗^*P* < 0.001, by *ANOVA* and Tukey's Multiple Comparison Test.

RNA isolated from the same transfected cells used for protein expression analysis was quantified by qRT-PCR. The relative mRNA values were standardized to reference ACTB. A significant difference of *ABCB4* mRNA expression was observed in *ABCB4*:c.1195G>C cells compared to the *ABCB4* wild-type cells (Figure 4(a)). Moreover, Western blot results also confirmed that *ABCB4* mutant V399L had significantly decreased MDR3 expression as compared to that of wild-type *ABCB4* and established mutant I541F (Figures 4(b) and 4(c)). The expression level of R652G was comparable with that of the wild type, whereas S346I and T424A showed a little decrease in expression because of stability and activity defect (previously reported) [21].

**Figure 5:**
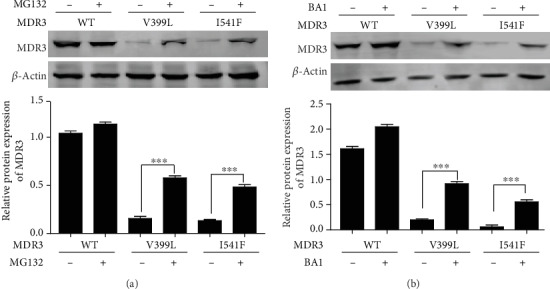
Effect of MG132 and bafilomycin A1 on MDR3 expression. MDR3 expression was examined in HEK-293T cells after transfection with *ABCB4* wild-type and mutant plasmids. Western blot was performed after 24 hr treatment with MG132 (a) or bafilomycin A1 (b). Data shown represent mean of three independent experiments and ^∗∗∗^*P* < 0.001 analyzed by *ANOVA* followed by Tukey's Multiple Comparison Test.

Based on impaired localization, we suspected that V399L mutant underwent folding or trafficking defect. Trafficking defective proteins usually undergo proteasomal or lysosomal degradation. We observed that after treatment with proteasomal inhibitor MG132 (Figure 5(a)) and lysosomal inhibitor bafilomycin A_1_ (Figure 5(b)), MDR3 expression of V399L, like I541F, was significantly increased. These results indicate that decreased MDR3 expression of mutant V399L might be a result of proteasomal or lysosomal degradation because of mistrafficking and intracellular retention.

### 3.6. Effect of Cyclosporin A and Low Temperature Could Rescue Trafficking Defective Mutant

**Figure 6:**
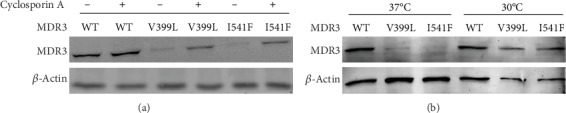
Effect of cyclosporin A and reduced temperature on ABCB4-WT and mutants. (a) HEK-293T cells expressing ABCB4-WT and mutants were treated with 10 *μ*mol/L cyclosporin A for 24 hours. (b) HEK-293T cells were grown at 37°C for 24 hr and shifted to 30°C after transfection. Cells were processed for electrophoresis and immunoblotting with the anti-MDR3 P3II-26 antibody.

The effect of cyclosporin A was checked on the expression and function of trafficking-defective mutant. Previous studies [19, 22] showed that trafficking-defective mutants of MDR3 were functionally rescued by cyclosporin A. We examined whether a trafficking-defective mutant of *ABCB4:c.1195G>C:p.V399L* could be rescued by cyclosporin A. After treatment with 10 *μ*M cyclosporine A, a substantial increase in MDR3 expression of V399L was observed (Figure 6(a)). Hence, our findings support the evidence that cyclosporin A significantly increased the expression of trafficking defective mutants by increasing the transport activity.

Previously, it was observed that most of the trafficking defective mutants in transporter proteins like MDR3 are temperature sensitive. Our results also found that the expression of trafficking defective mutants on the plasma membrane could be rescued by lowering temperature (Figure 6(b)).

### 3.7. ACMG Evaluation for Variant ABCB4:c.1195G<C:p.V399L

ACMG guidelines were followed for evaluating the pathogenicity of the identified variant. According to classification, one strong evidence of pathogenicity indicates that the variant *ABCB4:*c.1195G>C:p.V399L has well-established *in vitro* or *in vivo* functional studies supporting the damaging effect of gene variant on gene or by product (PS3). Two guiding evidences that indicate moderate pathogenicity are as follows: the variant is in a mutational hotspot or well-established functional domain (PM1) and the variant is absent from controls or population databases (ExAC, ESP, and TGP) (PM2). Three key points of evidence supporting pathogenicity are cosegregation with disease in affected family members in a gene known to cause disease (PP1), missense variant in a gene with a low rate of benign variation and has a role in common mechanism of disease (PP2), and patient's phenotype highly specific for disease with single gene etiology (PP4) (also supported the variant as “pathogenic”). Thus, *ABCB4:*c.1195G>C:p.V399L missense variant has one strong, two moderate, and three supportive guiding evidences supporting pathogenicity, fulfilling the criteria of ACMG for “pathogenic” variant.

## 4. Discussion

In the present study, we report a 13-year-old girl with a novel biallelic mutation of *ABCB4*, who experienced an intrahepatic cholestasis disorder of unknown etiology. Clinical examination and laboratory findings revealed a markedly elevated level of GGT. Based on WES and genetic screening of *ABCB4*, the patient was diagnosed with PFIC3. Genetic analysis followed by Sanger sequencing showed the patient is homozygous for a novel missense mutation V399L (Figure 2(a)). Various *in silico* tools also predicted the variant to be pathogenic and involved in the disruption of normal function and expression of MDR3 protein. Bioinformatics tools played an important role in evaluating the impact of mutation on protein structure, function, and conformation and interactive kinetics. According to HGMD (http://www.hgmd.org/) [23], up till now, more than 202 different types of mutations for biliary associated diseases have been reported in *ABCB4* and over 55 variants are found to be associated with PFIC3 (Supplementary Figs. 3A and B).

Different types of pathogenic mutations in *ABCB4* (≥70% missense) are associated with distinct clinical outcomes and result in a different cumulative risk. On the basis of functional characterization, different missense variants have been found to differently affect the function, subcellular localization, and expression of MDR3 protein. This results in misdiagnosis and complicates the treatment strategies for mutation carriers. Some missense variants result in impaired trafficking of MDR3 to the apical membrane because of improper folding and intracellular retention in the ER, which in turn will be degraded by endoplasmic reticulum-associated degradation (ERAD). Some mutations caused reduction in PC-translocating activity while some mutants normally processed, expressed, and successfully targeted to destination site, but their stability was affected because protein failed in binding to specific substrate. Patients carrying *ABCB4* mutations, that do not interrupt the folding or trafficking of MDR3 to the apical membrane, have mild PFIC3 phenotypes and can respond to chronic UDCA administration [24]. Such mutations also affect MDR3 function and activity resulting in decreased expression to some extent but do not impair the PC translocation pathway. Mainly, homozygous *ABCB4* mutations disrupt both the expression and normal trafficking of MDR3 leading to intracellular retention, premature degradation, and defective translocation pathway that negatively regulates PC transport activity. Patients with such mutations have severe phenotypes of progressive intrahepatic cholestasis that failed to respond to UDCA treatment and are at a risk for a liver transplantation. Previous studies reported that patients with monoallelic missense mutation in *ABCB4* have less severe phenotypes and progression of disease than biallelic missense or truncated mutations causing LOF and instability of *ABCB4* mRNA [5]. It was also found that mutations localized in transmembrane domain may effect substrate specificity while those found in conserved amino acid sequences, particularly near the walker motifs (A and B), may disrupt the floppase activity of MDR3 protein [25–30]. A previous study classified missense variations *via* functional characterization based on differently affecting proteins, whether by disturbing maturation, activity, stability, or having no detectable defect.

On the basis of previous classification by Delaunay et al. [21], the functional characterization study was conducted to classify missense variant V399L identified in this study. Delaunay et al. classified nonsense and frameshift variants into class I, maturation defective variants in class II, variants affecting transport activity in class III, variants causing stability defect in class IV, and variants having no detectable effect on protein into class V [21]. We selected previously established variants from each class (I541F-II, S346I-III, T424A-IV, and R652G-V) and performed functional classification *via* expression and subcellular localization. We found that our variant (V399L) showed significantly reduced expression and intracellular retention like I541F-II. Our mutation was found to be localized near conserved amino acid sequences of walker A motif in ICD3 of first NBD, where two missense variants (Y403H [31] and T424A [5]) have already been reported for PFIC3. Previously reported variant I541F is also located near signature motif sequence of NBD1.

Our results also found that reduced expression of V399L might be due to proteasomal and lysosomal degradation while being retained intracellularly. Previously, it was observed that targeting defect and protein degradation of certain mutants retained in the ER had improper folding. Misfolded or partially folded proteins, being inactive, are recognized by specialized chaperons and degraded by quality control machinery of the cell, ensuring that only properly folded proteins checkout to their target site. Functionality of defected trafficking and misfolded mutant protein could be restored by pharmacological modulators or lowering of intracellular temperature. We observed that translocation, expression, and the maturation of trafficking defective mutants were restored by pharmacological chaperon, cyclosporin A, and by the alteration of temperature treatment. It was observed from previous studies that if trafficking defective mutant protein could be rescued properly, then it will not affect the activity or stability of native protein.

In our case, the patient's parents are asymptomatic and neither have any family history of liver-associated disorders. However, one of the elder sisters of the patient died at the age of 10 because of misdiagnosis of a disease likely having the same symptoms. Our patient was diagnosed with PFIC3 based on WES, genetic analysis, and Sanger sequencing of *ABCB4* that confirmed a homozygous missense mutation. Biochemical laboratory reports also confirmed the diagnosis as PFIC3 because of a high level of GGT as compared to other types of PFIC where the GGT level is normal. By utilizing next-generation sequencing technologies, previous genetic studies identified different genes responsible for five types of PFIC. On the basis of functional characterization studies, different types of PFIC were found to be related to mutations in the hepatocellular transport system [32].

## 5. Conclusion

In conclusion, PFIC3 (rare) is difficult to diagnose and is sometimes misdiagnosed with other liver diseases due to lack of genetic screening and counseling for the identification of underlying factors. WES and genetic screening play an important role in the identification and definitive diagnosis of pathogenic mutations. The identification of a novel *ABCB4* (c.1195G>C) mutation in a patient with PFIC3 is of high clinical significance and may contribute to widening the spectrum of *ABCB4* mutations. Our study not only provides genetic basis for linking mutation to be deleterious but also gives functional characterization *via* comparative studies. Our results also support the evidence that cyclosporin A significantly increases the expression of trafficking defective mutants.

## Data Availability

The WES data and all the related material used to support the findings of this study are available from the corresponding author upon request.

## Abbreviations

ABCB4: ATP-binding cassette subfamily B member 4
BA1: Bafilomycin A1
BCA: Bicinchoninic acid assays
CsA: Cyclosporin A
GGT: Gamma-glutamyltranspeptidase
MDR3: Multidrug resistance type 3
PFIC3: Progressive familial intrahepatic cholestasis type 3
PC: Phosphatidylcholine
PCR: Polymerase chain reaction
THBA: Tetrahydroxylated bile acids
UDCA: Ursodeoxycholic acid
WES: Whole exome sequencing.

## Supplementary Materials

Supplementary Materials Supplementary Figure 1: describes that all the *ABCB4* mutant were constructed *via* the site-directed fast mutagenesis system using site-specific primers and verified by Sanger sequencing. Supplementary Figure 2: illustrating multiple sequence alignment of sequences among different species, generated by MUSCLE version 3.6 and edited by Jalview version 2.10.5, showed that novel mutation *ABCB4*:c.1195G<C:p.V399L was physicochemically and evolutionarily conserved. Supplementary Figure 3: elucidates that according to HGMD (www.hgmd.org/) data, up till now, different types of mutations for biliary associated diseases have been reported in *ABCB4* and over 55 variants are found to be associated with PFIC3 disease. Supplementary Table 1: describes that site-specific primer sequences were designed for PCR and DNA sequencing of the identified candidate gene *ABCB4* (exon 11). Supplementary Table 2: defines that identified V399L variant and other established missense mutations (S346I, T424A, I541F, and R652G) of *ABCB4* were constructed *via* the site-directed fast mutagenesis system by using site-specific mutation primers generated according to kit protocol. Supplementary Table 3: illustrates the laboratory test results of patient reported at the time of admission, revealing elevation in the liver enzymes, i.e., total bilirubin, direct bilirubin, reticulocytes, gamma-glutamyltransferase (GGT), alanine aminotransferase (ALT), alkaline phosphatase (ALP), aspartate aminotransferase (AST), and bile acids in the plasma.
