# Supplementary material for: Evaluation of a Novel Missense Mutation in ABCB4 Gene Causing Progressive Familial Intrahepatic Cholestasis Type 3
Source: Dis Markers. 2020 Jun 15;2020:6292818. doi: 10.1155/2020/6292818 (PMC7315263; doi:10.1155/2020/6292818)
Supplement: Supplementary Materials — Supplementary Figure 1: describes that all the ABCB4 mutant were constructed via the site-directed fast mutagenesis system using site-specific primers and verified by Sanger sequencing. Supplementary Figure 2: illustrating multiple sequence alignment of sequences among different species, generated by MUSCLE version 3.6 and edited by Jalview version 2.10.5, showed that novel mutation ABCB4:c.1195G<C:p.V399L was physicochemically and evolutionarily conserved. Supplementary Figure 3: elucidates that according to HGMD (www.hgmd.org/) data, up till now, different types of mutations for biliary associated diseases have been reported in ABCB4 and over 55 variants are found to be associated with PFIC3 disease. Supplementary Table 1: describes that site-specific primer sequences were designed for PCR and DNA sequencing of the identified candidate gene ABCB4 (exon 11). Supplementary Table 2: defines that identified V399L variant and other established missense mutations (S346I, T424A, I541F, and R652G) of ABCB4 were constructed via the site-directed fast mutagenesis system by using site-specific mutation primers generated according to kit protocol. Supplementary Table 3: illustrates the laboratory test results of patient reported at the time of admission, revealing elevation in the liver enzymes, i.e., total bilirubin, direct bilirubin, reticulocytes, gamma-glutamyltransferase (GGT), alanine aminotransferase (ALT), alkaline phosphatase (ALP), aspartate aminotransferase (AST), and bile acids in the plasma. [file 6292818.f1.docx]

**Evaluation of a novel missense mutation in *ABCB4* gene causing progressive familial intrahepatic cholestasis type 3**

Komal Saleem^a,b,#^, Qingbo Cui^c,#^, Tahir Zaib^a,b^, Siqi Zhu^a,b^, Qian Qin^a,b^, Yusi Wang^a,b^, Jinxi Dam^d^, Wei Ji^a,b^, Peng Liu^a,b^, Xueyuan Jia^a,b^, Jie Wu^a,b^, Jing Bai^a,b^, Songbin Fu^a,b^, Wenjing Sun^a,b*^

^a^Laboratory of Medical Genetics, Harbin Medical University, Harbin 150081, China; ^b^Key Laboratory of Preservation of Human Genetics Resources and Disease Control in China (Harbin Medical University), Ministry of Education, China; ^c^Pediatric surgery, the Second Affiliated Hospital of Harbin Medical University; ^d^Michigan State University.

^#^ These authors contributed equally to this work.

**^*^ Correspondence author: Dr. Wenjing Sun, MD, Ph.D, Professor**

Laboratory of Medical Genetics, Harbin Medical University, 157 Baojian Road, Nangang District, Harbin 150081, China. [sunwj@ems.hrbmu.edu.cn](mailto:sunwj@ems.hrbmu.edu.cn).

**Tel**: +86-451-86674798, **Fax**: +86-451-86674798

**Supplementary files description:**

- Supplementary figure.1 describes that all the *ABCB4* mutant were constructed *via* site-directed Fast Mutagenesis system using site-specific primers and verified by Sanger sequencing.
- Supplementary figure.2 illustrating multiple sequence alignment of sequences among different species, generated by MUSCLE version 3.6 and edited by Jalview version 2.10.5, showed that novel mutation *ABCB4*:c.1195G<C:p.V399L was physicochemically and evolutionarily conserved.
- Supplementary figure.3 elucidates that according to HGMD ([www.hgmd.org/](http://www.hgmd.org/)) data, up till now different types of mutations for biliary associated diseases have been reported in *ABCB4* and over 55 variants are found to be associated with PFIC3 disease.
- Supplementary table.1 describes that site specific primer sequences were designed for PCR and DNA sequencing of the identified candidate gene *ABCB4* (exon 11).
- Supplementary table.2 defines that identified **V399L** variant and other established missense mutations (**S346I, T424A, I541F and R652G**) of *ABCB4* were constructed *via* site-directed Fast Mutagenesis system by using site-specific mutations primers generated according to kit protocol.
- Supplementary table.3 illustrates the laboratory test results of patient reported at the time of admission, revealing elevation in the liver enzymes i.e. total bilirubin, direct bilirubin, reticulocytes, gamma-glutamyltransferase (GGT), alanine aminotransferase (ALT), alkaline phosphatase (ALP), aspartate aminotransferase (AST), and bile acids in the plasma.

**Supplementary figure legends**


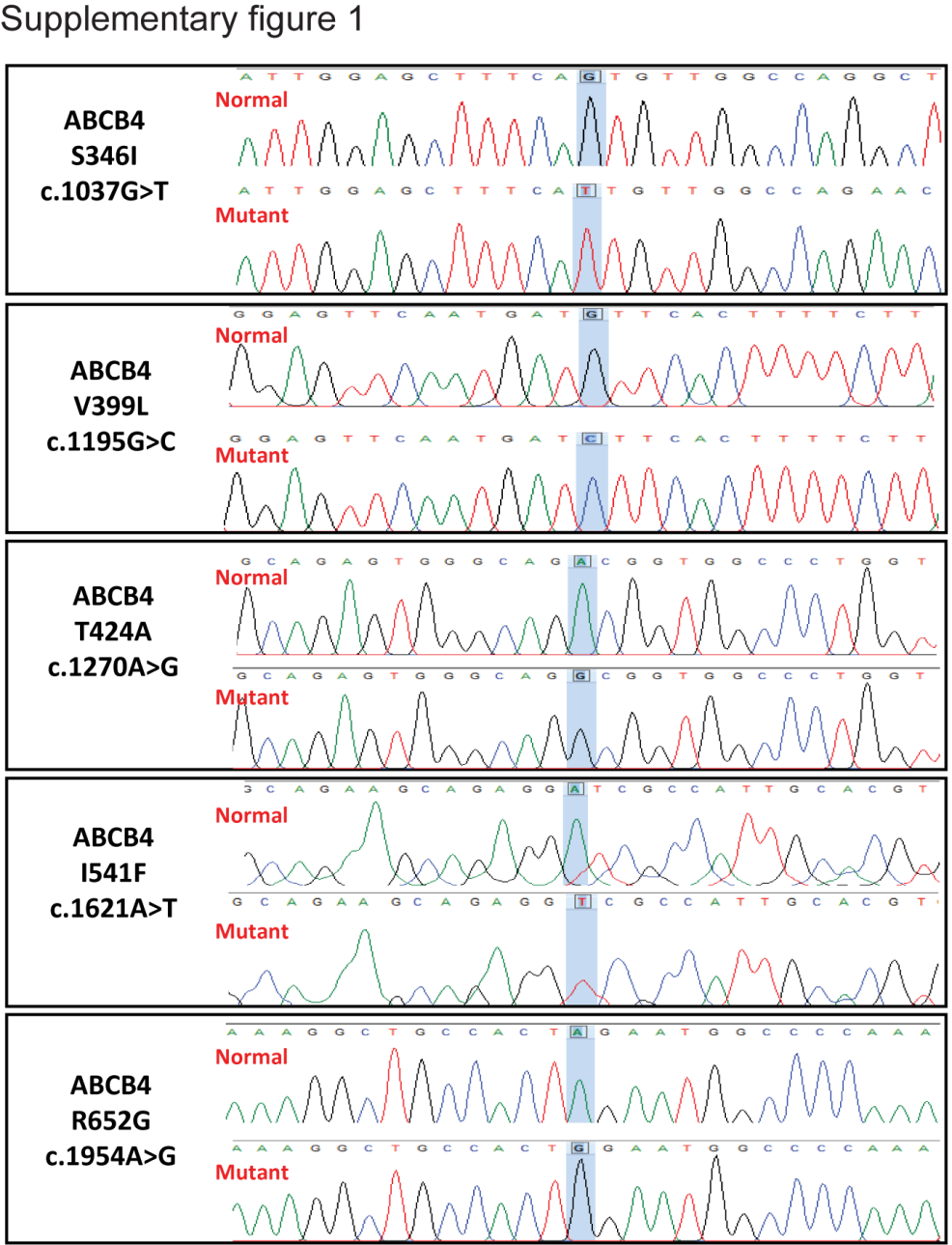


**Supplementary figure 1. Sanger sequencing results for verification of *ABCB4* mutant constructs.**


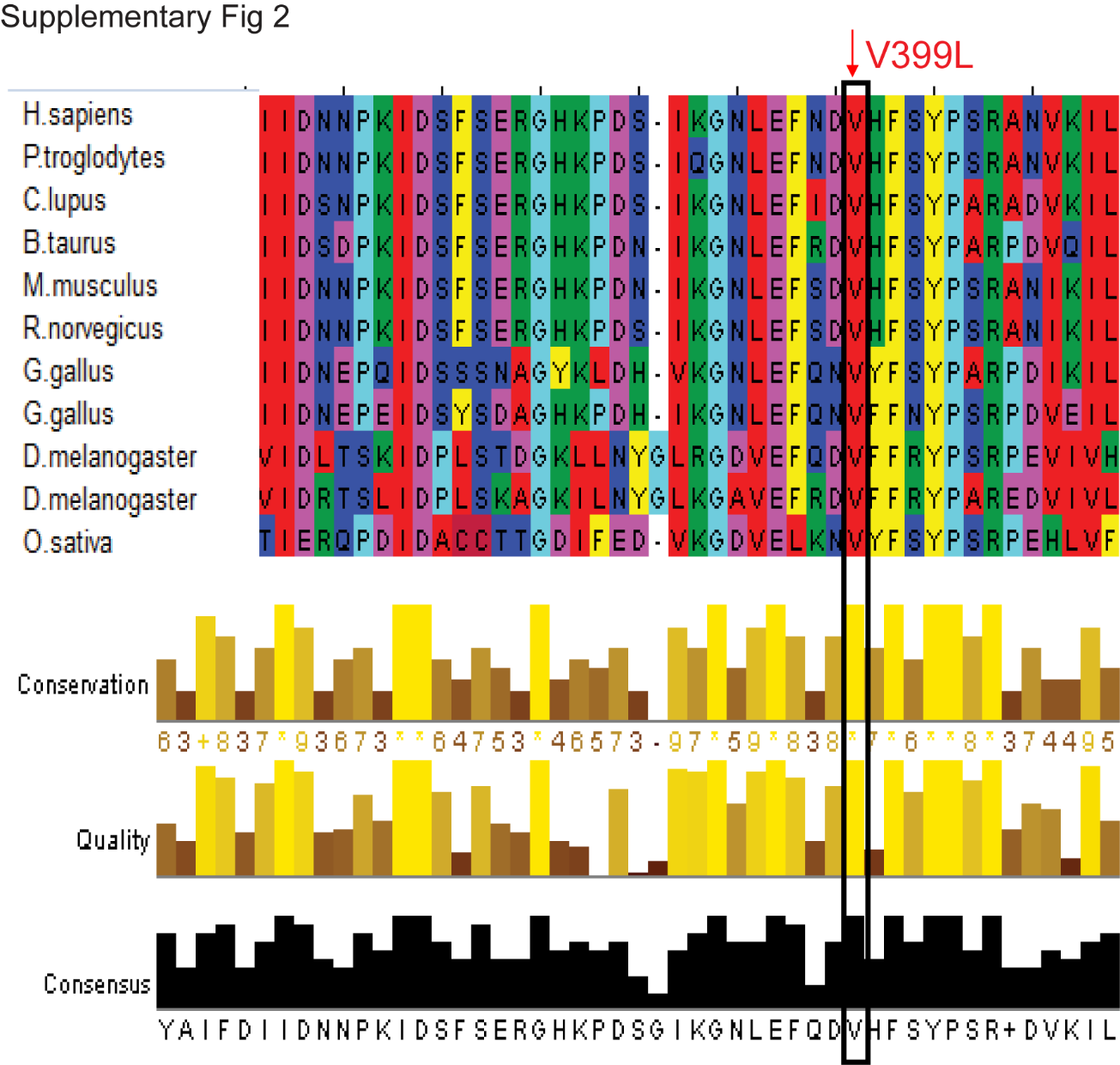
 **Supplementary figure 2.**  **Multiple sequence alignment generated by MUSCLE version 3.6 and edited by Jalview version 2.10.5.** Amino acids with related physicochemical properties have same color. Hydrophobic residues (I, V, L, A, M) are colored red, aromatic residues (F, W, Y) yellow, positive charged residues (K, R, H) green, negative charged residues (D, E) pink, hydrophilic residues (S, T, N, Q) blue, conformationally special (P, G) sky blue.


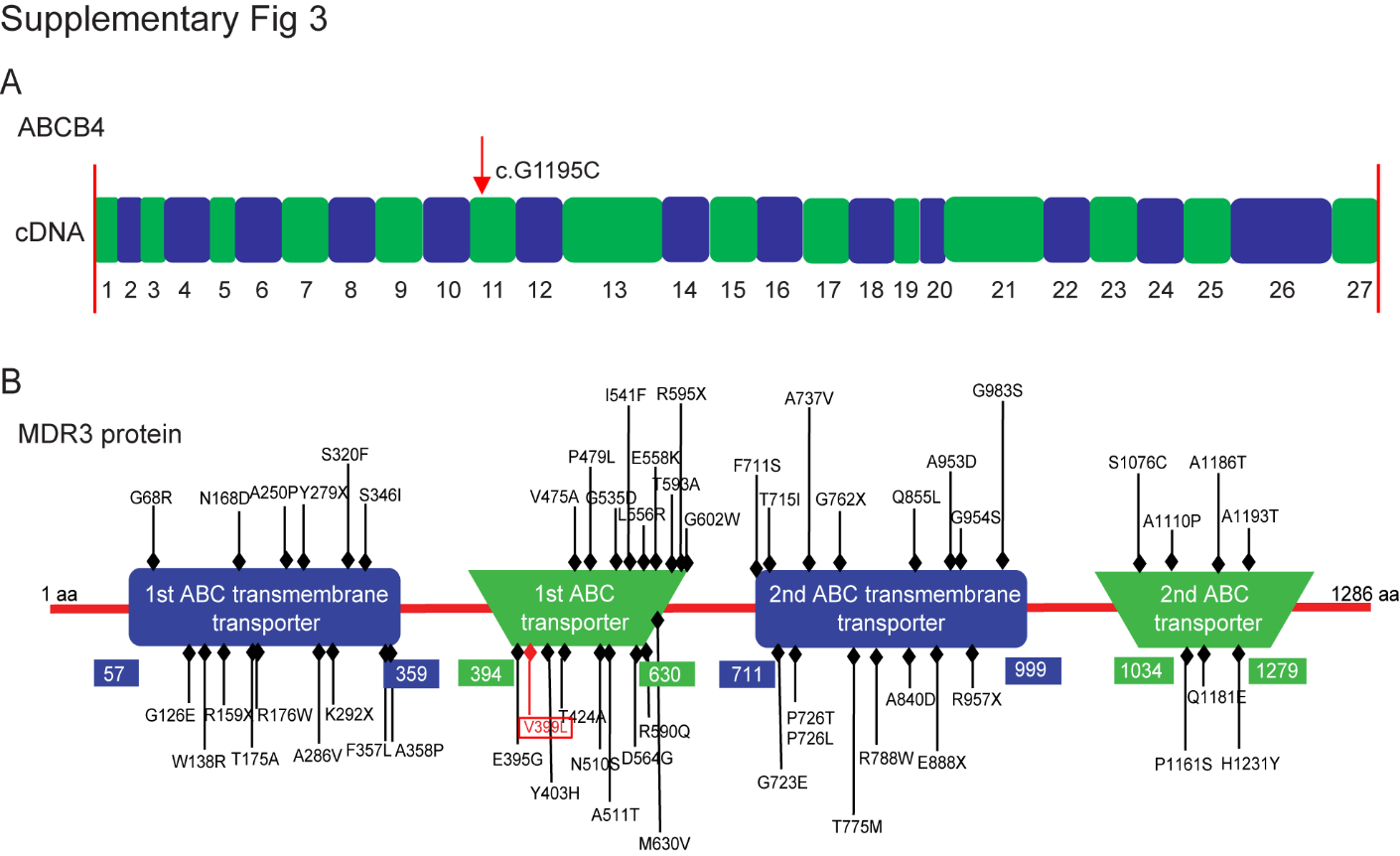


**Supplementary figure 3.**  **Structure of *ABCB4* gene and MDR3 protein product with an overview of reported mutations to date.** (A) Schematic representation of *ABCB4* gene depicting the identified missense variant. (B) Structure of MDR3 with different domains and location of different variants reported. Previously published mutations for PFIC3 are indicated in black and novel homozygous variant c.1195G>C identified in this study indicated in red in the 1^st^ ABC transporter domain. Numbers on the bottom of each domain indicate the amino acid position in MDR3 protein.

**Supplementary table legends:**

**Supplementary table 1. Primers used for PCR and Sanger sequencing**

| ***ABCB4*** | **Sequence** | **Tm (℃)** | **Amplicon (bp)** |
| --- | --- | --- | --- |
| **Forward** | 5'-CTTGTTTGTGCTATGATGGA-3' | 55.2**℃** | 204 |
| **Reverse** | 5-'ACAATCAACCTCAGTTAGG-3' | 55.2**℃** | 204 |

**Supplementary table 2. Mutagenic primers used to create mutant *ABCB4* plasmid constructs**

| **Mutant variants** | **Mutagenic primer sequences 5’-3’** | **Primer location** |
| --- | --- | --- |
| S346I c.1037G>T | Forward: AATTGGAGCTTTCATTGTTGGCCAG  Reverse: ATGAAAGCTCCAATTAGGATTGAAA | Exon 10 |
| V399L c.1195G>C | Forward: TGGAGTTCAATGATCTTCACTTTTC  Reverse: GATCATTGAACTCCAAATTCCCTTT | Exon 11 |
| T424A c.1270A>G | Forward: TGCAGAGTGGGCAGGCGGTGGCCCT  Reverse: CCTGCCCACTCTGCACCTTCAGGTT | Exon 12 |
| I541F c.1621A>T | Forward: GGCAGAAGCAGAGGTTCGCCATTGC  Reverse: ACCTCTGCTTCTGCCCACCACTCAG | Exon 14 |
| R652G c.1954A>G | Forward: AAAAGGCTGCCACTGGAATGGCCCC  Reverse: CAGTGGCAGCCTTTTCATCATTTAG | Exon 16 |

**Supplementary table 3. Biochemical laboratory results of patient**

| **Age of patient** | **13 years old (U/L)** | **Normal (U/L)** |
| --- | --- | --- |
| Total bilirubin (TB) (umol/L) | 61.3 | 10-30 |
| Direct bilirubin (DB) (umol/L) | 60.0 | 30-40 |
| Reticulocytes (Ret) | 3.4% | 0.5-2.5% |
| Gamma glutamyltransferase (GGT) | 280 | 9-48 |
| Alanine transaminase (ALT) | 125 | 7-56 |
| Alkaline phosphatase (ALP) | 381 | 20-140 |
| Aspartate aminotransferase (AST) | 187 | 10-40 |
| Red blood cells (RBC) | 3.38 | 4.5-5.5 |
| Hemoglobin (HGB) | 100 | 120-180 |
| White blood cells (WBC) | 3.9 | 4-10 |
| Lactate dehydrogenase (LDH) | 298 | 140-280 |
